# Consensus statement on the definition of orthostatic hypertension endorsed by the American Autonomic Society and the Japanese Society of Hypertension

**DOI:** 10.1038/s41440-022-01074-0

**Published:** 2022-11-22

**Authors:** Jens Jordan, Italo Biaggioni, Vasilios Kotsis, Peter Nilsson, Guido Grassi, Artur Fedorowski, Kazuomi Kario

**Affiliations:** 1 Institute of Aerospace Medicine, German Aerospace Center (DLR), Cologne, Germany; 2 Medical Faculty, University of Cologne, Cologne, Germany; 3 Autonomic Dysfunction Center and Division of Clinical Pharmacology, Department of Medicine, Vanderbilt University Medical Center, Nashville, TN USA; 4 3rd Department of Internal Medicine, Hypertension-24-h ABPM ESH Center of Excellence, Papageorgiou Hospital, Aristotle University of Thessaloniki, Thessaloniki, Greece; 5 Department of Clinical Sciences, Lund University, Malmö, Sweden; 6 Clinica Medica, Department of Medicine and Surgery, University of Milano-Bicocca, Milan, Monza Italy; 7 Department of Cardiology, Karolinska University Hospital, Department of Medicine, Karolinska Institute, Stockholm, Sweden; 8 Department of Cardiology, Jichi University School of Medicine, Tochigi, Japan

**Keywords:** Adrenergic, Autonomic nervous system, Baroreflex, Circadian rhythm, Orthostatic hypertension, Orthostatic pressor response, Sympathetic nervous system

## Abstract

We propose a consensus definition of “an exaggerated orthostatic pressor response” in subjects in whom systolic blood pressure increases ≥20 mmHg when going from the supine to standing posture. This definition can be extended for seated to standing measurements. We reserve the term “orthostatic hypertension” if this pressor response leads to an upright systolic blood pressure ≥140 mmHg. We believe this consensus definition will help in the study of the pathophysiology, clinical impact, and potential treatment of these entities, and the identification of patients that are at greater cardiovascular risk.

## Background

Standing imposes a major hemodynamic burden on the cardiovascular system. In healthy persons, neurohumoral reflex mechanisms, particularly the arterial baroreflex, maintain blood pressure with standing. However, the counterregulatory response overshoots in some people such that blood pressure increases with standing. This orthostatic pressor response may result in hypertensive blood pressure levels only when standing or further exacerbate arterial hypertension. The condition is often referred to as orthostatic hypertension and appears to be associated with increased cardiovascular risk [1, 2]. Yet, a uniform definition and diagnostic cutoff values of orthostatic hypertension have not been established. In fact, current hypertension guidelines do not cover orthostatic hypertension [3–5]. Moreover, diagnostic criteria for orthostatic hypertension vary from study to study. Therefore, data regarding the prevalence, cardiovascular risk, and management of orthostatic hypertension is difficult to interpret. We propose a new classification distinguishing between an exaggerated orthostatic pressor response and orthostatic hypertension to facilitate research in this area.

## Definitions

- An **exaggerated orthostatic pressor response** is a sustained increase in systolic blood pressure by at least 20 mmHg when changing from the supine to the standing position regardless of absolute blood pressure while standing.
- **Orthostatic hypertension** is defined as an exaggerated orthostatic pressor response associated with systolic blood pressure of at least 140 mmHg while standing.

## Rationale

Our goal was to arrive at a pragmatic definition that could be utilized in the setting of mechanism-oriented studies, epidemiological surveys, and clinical trials. We are aware that the suggested definitions are based on clinical reasoning and normative data rather than cardiovascular risk estimates and that the definitions as well as blood pressure cutoff values may have to be refined in the future. While orthostatic hypertension may be associated with cardiovascular risk as outlined in the sections below, more data is required to allow for a risk-based definition.

In the Malmö Preventive Project, blood pressure measurements after 10 min in the supine position and after one-minute standing were measured in 33,346 persons aged 45.7 ± 7.4 years [6]. On average, systolic blood pressure decreased 1.2 mmHg with a standard deviation of 8.8 mmHg. In the follow-up study to the Malmö Preventive Project, the Malmö Offspring Study [7], among 4019 participants aged 41.9 ± 14.5 years, systolic blood pressure increased by 1.4 ± 8.6 mmHg after 3 min of standing corroborating Malmö Preventive Project observations. Thus, 20 mmHg systolic blood pressure cutoff value for an exaggerated orthostatic pressor response corresponds to approximately two standard deviations above the population mean.

An increase in diastolic blood pressure on standing can be normal. Moreover, in individuals with excessive orthostatic heart rate increases, cardiac stroke volume may decrease such that pulse pressure is narrowed. The resulting spurious diastolic blood pressure increase is difficult to interpret. The panel, therefore, suggests that systolic blood pressure may be more practical when diagnosing orthostatic hypertension.

A recent publication by an expert group suggested that a systolic blood pressure increase while standing by at least 20 mmHg or above 140 mmHg in otherwise normotensive persons could serve as criteria for orthostatic hypertension [8]. The limitation of this broad definition is that individuals with upright blood pressure in the normotensive range could be labeled as being “hypertensive”. Even though we cannot exclude that orthostatic blood pressure surges even within the normotensive range can have negative influences on the cardiovascular system, it appears likely that an absolute standing blood pressure in the upright posture is more relevant to cardiovascular risk. Therefore, we propose to distinguish between an exaggerated orthostatic pressor response, which may result in normotensive or hypertensive blood pressure while standing, and factual orthostatic hypertension. Among persons with orthostatic hypertension, some may exhibit isolated orthostatic hypertension, which is an orthostatic pressor with hypertensive blood pressure values only while standing. Others may also be hypertensive while supine or seated with further worsening of blood pressure when standing. Possibly, these subgroups may require a different diagnostic and therapeutic approach [2, 9].

## Pathophysiology of orthostatic hypertension

With standing, approximately 500–1000 ml blood is pooled below the diaphragm and hydrostatic pressure shift fluids from the intravascular to the interstitial compartment. In healthy persons, compensatory autonomic reflex mechanisms withdraw cardiac parasympathetic drive and raise cardiac and vascular sympathetic activity thereby leading to an increase in heart rate, a drop in systolic blood pressure <20 mmHg, and a small increase in diastolic blood pressure [10, 11]. Small-scale studies suggest that orthostatic hypertension results from an excessive increase in vascular resistance with standing [12], however, the mechanisms driving orthostatic hypertension may be affected by age [13, 14]. The observations that plasma norepinephrine increases more with standing in patients with orthostatic hypertension suggest that the response is mediated through excessive sympathetic activation with standing [12, 15]. Indeed, alpha-adrenoreceptor blockade attenuates orthostatic hypertension [16]. Patients with orthostatic hypertension often feature an extreme dipping pattern in 24 h ambulatory blood pressure recordings [14], and an exaggerated early morning blood pressure surge [15].

## Epidemiology and cardiovascular risks of orthostatic hypertension

The epidemiology of orthostatic hypertension and the data regarding the cardiovascular risk associated with orthostatic hypotension have been reviewed recently [1]. Briefly, orthostatic hypertension defined as a sustained increase in systolic blood pressure ≥20 mmHg and/or diastolic blood pressure ≥10 mmHg within 3 min of standing was observed in 5–30% of participants in epidemiological surveys or clinical trials [17–21]. Risk factors for orthostatic hypertension included age, adiposity, arterial hypertension, and diabetes mellitus [22]. An exaggerated orthostatic pressor response with office blood pressure in the normotensive range may precede arterial hypertension in younger people [23]. Among people with arterial hypertension, individuals showing an exaggerated orthostatic pressor response are more likely to exhibit subclinical cerebrovascular disease, peripheral arterial disease, or cerebrovascular disease [15, 24–26]. Orthostatic hypertension was also associated with increased all-cause and cardiovascular mortality [17, 27, 28]. The clinical significance of these findings is difficult to generalize because in the past, diagnostic criteria for orthostatic were not harmonized.

## Diagnosing orthostatic hypertension

When diagnosing an exaggerated orthostatic pressor response or orthostatic hypertension, blood pressure measurements with a brachial cuff suffice. Pulse rate should also be recorded. Preferably, measurements should be conducted in a quiet surrounding to limit confounding of orthostatic blood pressure responses by external stimuli. Supine blood pressure and heart rate should be measured after five minutes of rest in the supine position. Then, patients should stand up actively. Upright blood pressure and heart rate should be measured after 1, 3, and 5 min, at least, to confirm the persistent character of the blood pressure change. We suggest that the measurements after 3 and 5 min be averaged to determine standing systolic blood pressure. For screening purposes, a single measurement after 3 min may be sufficient.

Beat-to-beat blood pressure and heart rate recordings can be applied in selected patients but are not widely available [8].

We recommend that repeated orthostatic testing should be conducted on a different day to confirm the diagnosis.

Because supine and upright blood pressure measurements may be difficult to conduct in all patients, seated to upright measurements may be considered for screening purposes. Since hemodynamic changes when standing up from the seated standing position are less compared with standing up from the supine position, the orthostatic pressor response may be less pronounced such that the sensitivity in detecting orthostatic hypertension may decrease. Similar limitations have been observed when using seated to standing measurements when diagnosing orthostatic hypotension [29]. Therefore, we suggest that confirmatory testing be considered with supine to standing measurements.

Head-up tilt testing may be considered for persons unable to undergo an active standing test.

## Clinical management

There is no evidence that individuals with an exaggerated orthostatic pressor response and blood pressure within the normal range require antihypertensive therapy. It may be prudent to follow up these patients’ blood pressure given the increased risk for progression to arterial hypertension. In patients with isolated orthostatic hypertension in whom blood pressure is only in the hypertensive range while standing, ambulatory blood pressure monitoring may have utility in gauging the overall blood pressure load during the day and detecting abnormalities in diurnal blood pressure patterns, such as extreme blood pressure dipping during the night or masked morning hypertension [2, 14]. Patients fulfilling orthostatic hypertension criteria have not been specifically sought for or excluded from blood pressure trials. It appears reasonable prescribing antihypertensive therapies according to current hypertension guidelines [3–5]. There is no evidence that patients with orthostatic hypertension benefit from a particular drug class in terms of cardiovascular risk protection. Whether diuretics, which could further increase neurohumoral activation while standing should be avoided in patients with orthostatic hypertension is unclear given the known benefits of such drugs on cardiovascular morbidity and mortality. Overall, there is a need for additional research to guide clinical decision making in patients with an exaggerated orthostatic pressor response and orthostatic hypertension. We hope that a uniform definition will support this endeavor.
